# Congenital malaria in Urabá, Colombia

**DOI:** 10.1186/1475-2875-10-239

**Published:** 2011-08-16

**Authors:** Juan G Piñeros-Jiménez, Gonzalo Álvarez, Alberto Tobón, Margarita Arboleda, Sonia Carrero, Silvia Blair

**Affiliations:** 1 Malaria Group, Universidad de Antioquia, Calle 62, # 52-59, Laboratorio 610, Medellín, Colombia; 2 Doctorado Interfacultades en Salud Pública, Universidad Nacional de Colombia, Bogotá, Colombia; 3 Instituto de Medicina Tropical Antonio Roldán Betancur-CES, Apartadó, Colombia

## Abstract

**Background:**

Congenital malaria has been considered a rare event; however, recent reports have shown frequencies ranging from 3% to 54.2% among newborns of mothers who had suffered malaria during pregnancy. There are only a few references concerning the epidemiological impact of this entity in Latin-America and Colombia.

**Objective:**

The aim of the study was to measure the prevalence of congenital malaria in an endemic Colombian region and to determine some of its characteristics.

**Methods:**

A prospective, descriptive study was carried out in the mothers who suffered malaria during pregnancy and their newborns. Neonates were clinically evaluated at birth and screened for *Plasmodium spp*. infection by thick smear from the umbilical cord and peripheral blood, and followed-up weekly during the first 21 days of postnatal life through clinical examinations and thick smears.

**Results:**

116 newborns were included in the study and 80 umbilical cord samples were obtained. Five cases of congenital infection were identified (four caused by *P. vivax *and one by *P. falciparum*), two in umbilical cord blood and three in newborn peripheral blood. One case was diagnosed at birth and the others during follow-up. Prevalence of congenital infection was 4.3%. One of the infected newborns was severely ill, while the others were asymptomatic and apparently healthy. The mothers of the newborns with congenital malaria had been diagnosed with malaria in the last trimester of pregnancy or during delivery, and also presented placental infection.

**Conclusions:**

Congenital malaria may be a frequent event in newborns of mothers who have suffered malaria during pregnancy in Colombia. An association was found between congenital malaria and the diagnosis of malaria in the mother during the last trimester of pregnancy or during delivery, and the presence of placental infection.

## Background

Most neonatal and perinatal deaths are preventable. According to The State of the World's Children report, published in 2009, 3.7 million children die annually during the first 28 days of postnatal life, 75% during the first week and 36% due to severe infection [1]. Malaria continues to be one of the most important public health problems worldwide, and is responsible for 8% of the mortality among children below the age of five [1]. *Plasmodium spp*. is usually transmitted by *Anopheles spp*. mosquitoes, but can also be transmitted from mother to child causing congenital malaria (CM) [2]. Maternal and placental infections caused by *Plasmodium spp*. are frequent entities in malaria endemic regions and have been extensively studied in Africa, Asia and Oceania; in contrast, there is little information regarding CM. The first studies by Covell and Bruce-Chwatt in the midst of the 20^th ^century reported an incidence of CM between 0.18% and 0.3% in newborns from mothers suffering from malaria during pregnancy in hyper-endemic regions [3,4], and only 300 sporadic cases were reported worldwide in the literature during the next half century; therefore, CM has been considered a rare event [5]. However, since 1985 there have been reports of a CM incidence ranging from 4.9% to 54.2% in regions of Africa with stable malaria transmission, where it is more likely that the pregnant women have acquired immunity to prevent mother-to-child transmission [6-11]. The few reports in Latin America include some cases from Brazil, Mexico and Honduras [12-15], and a case-control study that reported a congenital infection incidence of 3.3% [16]. In Colombia, 12 cases were reported before 1986 [17] and there is a recent report on five cases of severe neonatal malaria caused by *Plasmodium vivax*, one of them with congenital infection based on epidemiological criteria [18]. Because no studies have been carried out to establish the magnitude of CM in malaria endemic regions in Colombia, this study was designed to evaluate the prevalence of CM in a high-endemic region in Colombia and to describe its clinical and epidemiological characteristics.

## Methods

### Study location

This investigation was carried out in the Caribbean region of Urabá (Department of Antioquia, Colombia). This region is close to the Panama border, with tropical rainforest ecosystems and a population of 509,409 people, most of them African descendants (51.5%) and under 25 years of age (59.0%). Banana growing and cattle farming are the main economic activities in this region [19]. The study was conducted in the municipalities of Apartadó (7° 52' 40'' N; 76° 37' 44'' W), Carepa (7° 45' 12''N; 76° 39' 21''W), Turbo (8° 05' 42''N; 76° 44' 23''W) and Necoclí (8° 25' 39''N; 76° 46' 58''W). The annual incidence of malaria in the region was higher than 40 cases per 1,000 inhabitants during 1998-2007, with unstable transmission, and where more than 75% of reported cases were caused by *P. vivax *[20]. An incidence of 4.1 cases of gestational malaria for each 1,000 live births has also been reported in this region [21].

### Study design and study population

This was a prospective, descriptive study that was part of a larger epidemiological study on gestational malaria that included 2,117 pregnant women and was carried out from 2005-2007 by the Malaria Group of the Universidad de Antioquia in Colombia. Pregnant women were screened for *Plasmodium spp*. infection by thick smear at each prenatal visit and during delivery, and/or when they consulted due to a febrile syndrome. At birth, newborns from mothers who had a diagnosis of malaria in pregnancy (MIP) were clinically assessed and thick smears from peripheral blood and umbilical cord were analysed for the presence of the parasite. Weekly clinical and parasitological follow-up was carried out for 21 days in newborns, according to the criteria proposed by Henrys for CM in unstable transmission areas [22], such as this Latin-American region. The units of analysis were the newborns or the umbilical cords of mothers who had suffered MIP.

### Sample size

The appropriate sample size was calculated [23] based on an annual average of 11,000 births [24], with a 10% estimated frequency of MIP (n = 1,650 newborns from mothers with MIP), and a 7% expected frequency of CM. The calculated sample size was 95 units of analysis, and we added 20% more to make up for possible losses during the follow-up period. A total of 116 newborns were finally selected according to the following inclusion criteria: 1) child of a pregnant woman who had been diagnosed with Plasmodium infection during prenatal control and/or during delivery, 2) newborn residing in the municipalities where the study was carried out, and 3) informed consent from the mother or guardian for the newborn's participation in the study.

### Definitions

*Congenital malaria: *1) newborn of a MIP mother with an infection caused by the same *Plasmodium *species at birth or during the 21-days follow-up period or 2) *Plasmodium spp*. infection of the umbilical cord [18].

*Adolescent: *under 20 years of age [25].

*Low birth weight (LBW): *less than 2,500 g [26].

*Preterm birth (PB): *birth before 37 weeks of gestation [26].

*Small for gestational age: *below the 10^th ^percentile of weight for gestational age, according to Lubchenco curves [26].

*Gestational age: *determined by ultrasound or the first day of the mother's last menstrual period.

### Thick smears

#### Peripheral blood thick smear

capillary puncture was performed and the first drop of blood was discarded; the following drops were used for diagnosis [27]. Thick smears were stained with Field stain and read under a light microscope at 100× magnification. Parasitaemia was estimated against 200 leukocytes (8,000 leukocytes/μL, standard value) and was expressed as parasites/μL. *P. falciparum *parasitaemia was calculated counting ring forms, while *P. vivax *parasitaemia was calculated counting all asexual forms. A sample was considered negative if after the examination of 200 microscopic fields, no parasites were observed or when only *P. falciparum *gametocytes were observed [28].

#### Umbilical cord thick smear

1 mL of blood was extracted from the umbilical vein during delivery [23]. Two thick smears were made and analysed using the same technique previously described [28].

#### Placental blood thick smear

placentas were cleaned with 50 mL of 0.9% saline solution. A 1-cm^3 ^incision was made in a cotyledon close to the umbilical cord, from where placental blood samples were collected [29].

### Data collection

Newborn data from birth, physical exam and anthropometric measurements were registered in a standardized form (Latin-American Centre for Perinatology) [30]. Registered variables were gender, birth weight, height, cephalic perimeter, and the APGAR score at one and five minutes after birth. Newborn health status was evaluated by a physician on a weekly basis, and thick blood smears were made from peripheral blood.

### Statistical analysis

Statistical analyses were made with EpiInfo software, version 6.04 (Center for Disease Control and Prevention, USA; World Health Organization, Geneva, Switzerland). A descriptive analysis was made to summarise and present the information. Median and interquartile range (IQR) or mean and confidence interval were calculated for continuous variables (according to normal distribution of data), and absolute frequency and percentages were used for categorical variables. Mann Whitney's test and chi-squared test were used to compare maternal and neonatal characteristics between the newborn groups with and without CM. A significance level of 5% was chosen.

### Ethical considerations

The project was approved by the Ethics Committee of the Faculty of Medicine, Universidad de Antioquia. The Colombian Ministry of Health research standards (resolution 008430/1993), the WHO ethical standards for research on human beings and the 2002 Helsinki declaration were taken into account. Informed consent was obtained from all the mothers of the newborns or from their legal guardians.

## Results

### Findings in the mothers and newborns

From a total of 220 pregnant women who were diagnosed with MIP during prenatal control, only 157 gave birth in a health facility and were accessible; 116 of them were selected to be included in the study after fulfilling the criteria. The general characteristics of the mothers and newborns included (n = 116) and not included (n = 41) in the study are shown in Table 1. The only statistically significant difference between the mothers included and not included in the study was found in the median of years of residence in endemic areas (p = 0.024), the other characteristics were similar.

**Table 1:** General characteristics of the mothers and newborns included and not included in the study

Variable	Cases included in the study		Cases not included in the study		Mann-Whitney p value
		
	n	Q2 (Q1 - Q3)	n	Q2 (Q1 - Q3)	
Maternal age	115	21 (18 - 27)	40	22 (18 - 28)	0,845

Maternal haemoglobin after week 20 (g/dL)	80	11.5 (10.1-2.0)	21	11.6 (10.7 - 11.6)	0,855

Years of residence in malaria area	114	18 (10 - 23)	41	15 (5 - 20)	0,024

Previous pregnancies	116	2 (1-3)	41	1 (1-3)	0.91

Episodes of malaria in the previous year	78	0 (0 - 1)	33	0 (0 - 1)	0,347

Gestational age at birth	109	38.4 (37.8 - 39.7)	38	39 (37.7 - 40)	0,055

Neonatal weight	115	2875 (2500 - 3425)	39	3005 (2800 - 3400)	0,280

Neonatal length	116	48 (47 - 50)	40	49 (47 - 50)	0,697

APGAR at 1 minute	112	8 (7 - 9)	40	8 (8 - 9)	0,616

Episodes of malaria during current pregnancy	116	1 (1 - 1)	41	1 (1 - 2)	0,166

Q2: median Q1-Q3: quartil 1-quartil 3.

37.9% of the mothers included in the study were adolescents and 6.4% were over 35 years of age; 21.8% were primigravida, 19.5% reported 3 or more previous pregnancies, and 69.8% had been living in malaria risk areas for 10 years or longer. 91.4% of the pregnant women were attending a prenatal care program with a median of 4 visits, 34.5% reported having at least one episode of malaria in the previous year, and 12.7% had PB; 9.1% of the newborns had LBW and 18.2% were small for gestational age.

One hundred and sixty-eight episodes of malaria (138 caused by *P. vivax *and 30 by *P. falciparum*) were diagnosed among the 116 mothers (average, 1.4 episodes per mother). The median for the gestational age at the time of diagnosis of the first malaria episode was 30.1 weeks (IQR: 22.2-35.8). Twenty-seven episodes of malaria were diagnosed in the mothers during delivery, 21 caused by *P. vivax *and six by *P. falciparum*; the median parasitaemia was 2,400 parasites/μL (IQR: 400-5,560).

### Findings in the placenta

Ninety-four placental blood samples were obtained from the 116 mothers; 17 (18%) had *Plasmodium *infection (14 *P. vivax *and three *P. falciparum*) with a median parasitaemia of 200 parasites/μL (IQR: 80-920). Two mothers who were reported as negative for *Plasmodium *infection in peripheral blood samples during the prenatal control and delivery, had parasites in the placentas (one *P. vivax *and one *P. falciparum*).

### Congenital malaria prevalence

Of the 80 blood samples collected from the umbilical cords at birth, two (2.5%) were positive for *P. vivax*. The 116 newborns were followed-up for 20.5 ± 1.1 days with an average of 2.8 ± 1 postnatal controls per neonate. During follow-up, three (2.6%) neonates were diagnosed with *Plasmodium spp*. infection; two by *P. vivax *(one at birth and one on day 14), and one by *P. falciparum *(on day 8). CM was diagnosed in five cases, for a prevalence of 4.3%, three diagnosed in the 116 newborns and two in the 80 umbilical cords. None of these results were positive in both units of analysis simultaneously.

### Characteristics of mothers and newborns with and without CM

There were no significant differences in the characteristics of mothers of newborns with and without CM (p > 0.05) (Table 2). The median age of mothers with newborns with CM was 23 years (IQR: 21.5-31), had a median of 1.0 for previous pregnancies (IQR: 1.0-3.5), and had attended at least four prenatal controls. The median episodes of malaria during the current pregnancy was 1.0 (IQR: 1.0-1.5), and the median gestational age at the time of maternal malaria diagnosis was 30 weeks (IQR: 25.6-39.4). The median gestational age of the mothers of CM cases was 37.7 weeks (IQR: 37.3-40.3).

**Table 2:** Maternal and neonatal characteristics of congenital and non-congenital malaria groups

Variables	Congenital malaria (n = 5)	Non congenital malaria (n = 111)	p value
Maternal characteristics			
Maternal age (years), median (Q1-Q3)	23 (21.5-31)	22 (18-28)	0.550
Previous pregnancies, median (Q1-Q3)	1 (1-3.5)	1 (1-3)	0.676
Years of residence in endemic areas, median (Q1-Q3)	22 (10.5-28)	15 (5-19.5)	0.135
Episodes of malaria in the previous year, median (Q1-Q3)	0 (0-0)	0 (0-1)	0.340
Episodes of malaria during current pregnancy, median (Q1-Q3)	1 (1-1.5)	1(1-2)	0,476
Gestational age at the time of malaria diagnosis (weeks), median (Q1-Q3)	30 (25.6-39-4)	30.3 (21.5-35.4)	0.662
Arterial hypertensive disorders during pregnancy, (%)	25	6.9	0.275*
Urinary tract infections, (%)	0	26.3	0.570*
Preterm birth, (%)	20	11.3	0.470*
Neonatal characteristics			
Weight (grams), median (Q1-Q3)	3000 (2800-3400)	3200 (2200-3475)	0.633
Length (cm), median (Q1-Q3)	48 (44-49.5)	49 (47.5-50)	0.406
Cephalic perimeter (cm), median (Q1-Q3)	34 (31.5-34)	34 (33-34)	0.238
APGAR at 1 minute, median (Q1-Q3)	8 (5.5-9)	8 (8-9)	0.477

*Fisher's exact test, Q1-Q3: quartil 1-quartil 3.

There were no significant differences in the anthropometric characteristics and neonatal adaptability among neonates with and without CM (Table 2). Medians of weight, length and cephalic perimeter at birth of CM cases were 3,000 g (IQR: 2800-3400), 48 cm (IQR: 44-49.5) and 34 cm (IQR: 31.5-34), respectively; all these measurements were above the 10^th ^percentile according to the Lubchenco curves.

The mother of the congenital case caused by *P. falciparum *presented clinical and laboratory signs compatible with severe malaria (parasite count of 106,640 ring forms/μL and spontaneous bleeding at venopuncture sites), and required hospitalization in a high complexity health institution in the immediate postpartum period. The mothers of the other CM cases, caused by *P. vivax*, were classified as having acute non-complicated malaria; three were diagnosed at the time of delivery and had concomitant placental infection, and one was diagnosed in the third trimester of pregnancy during the prenatal control (Table 3). All mothers and infected newborns received anti-malarial treatment according to the national protocols [31].

**Table 3:** Malaria and thick smear findings in CM cases

	Case 1	Case 2	Case 3	Case 4	Case 5
Malaria findings					
Episodes of maternal malaria	1 *Pv *	1 *Pv* 1 *Pf*	1 *Pv*	1 *Pv*	1 *Pf*
Gestational age at malaria diagnosis (weeks + days)	37 + 2	*Pf*: 24 + 2 *Pv*: 40 +2	27 + 6	40 + 4	30
Type of malaria syndrome in mothers	acute uncomplicated malaria	acute uncomplicated malaria	acute uncomplicated malaria	acute uncomplicated malaria	severe malaria
Clinical status of neonates at the time of congenital malaria diagnosis	afebrile	afebril	afecrile	afebrile	severely ill
Thick smear findings					
Maternal blood during delivery	*Pv *(nd)	*Pv *(560)	Neg.	*Pv *(120)	*Pf *(106640)
Placental blood	*Pv *(920)	*Pv *(440)	Neg.	*Pv *(80)	Neg.
Umbilical cord blood	*Pv *1860)	Neg.	Neg.	*Pv *(80)	Neg.
Neonatal blood	Neg.	*Pv *(40) day 1	*Pv *(160) day 14	Neg.	*Pf *(120) day 8

Pv: *Plasmodium vivax*, Pf: *Plasmodium falciparum*

( ) parasite/μL.

Newborns with *P. vivax *infection in the umbilical cord (Table 3, case 1 and case 4) did not show infection in peripheral blood during follow-up or manifested clinical signs of active infection or severe disease; thus, no anti-malarial treatment was administered. The *P. falciparum *CM case was severely ill since birth due to prematurity and to hyaline membrane disease (Table 3, case 5). During hospitalization in a neonatal intensive care unit, the newborn was diagnosed with early neonatal sepsis by *Escherichia coli*. The thick smear was negative at birth, but serial thick smears started on day 6 revealed *P. falciparum *infection on day 8 (120 rings/μL).

## Discussion

CM is one of the least known and studied adverse events of MIP [2]. The present study is the first epidemiological report about CM in Colombia and could be considered as being one of the first for regions where *P. vivax *malaria is the most frequent specie. This study found a prevalence of CM of 4.3% among newborns from mothers with MIP in the Urabá region. Such frequency was similar to that found in Nigeria by Falade *et al*, who identified a 5.1% parasitaemia in samples from newborns whose mothers had suffered from MIP [11], and to the prevalence of 3.3% (1/30) found by Fernández *et al *in Honduras in 2001 [16], but greater than that reported by Alves *et al *in Brazil, who found just one case of CM in 2,781 cases of malaria between 1980 and 1994 in the Campinas region [15]. 80% (four out of five) of CM cases in this study were caused by *P. vivax*, the Plasmodium responsible for most of the malaria cases in the Americas [32].

The umbilical cord and newborns were the units of analysis for obtaining such prevalence, and the first 21 days of life were defined as the time limit for obtaining samples. Although in *P. falciparum *hyper-endemic regions the first seven days of life are the limit to define an infection as congenital, we chose a time limit that has been proposed for areas of low endemicity and unstable transmission, which is an accepted limit for infections caused by non *P. falciparum *species [22]. One of cases was identified at postnatal day14, within the time limit, however it is not possible to guarantee that it was not caused by vector transmission of the parasite because we had no information on the control measures used to protect this neonate against malaria; the question remains on whether it was a postnatal infection, however if we consider the unstable and low malaria transmission rates of the study location, is not likely that this occurred. The CM case by *P. falciparum *was diagnosed at postnatal day 8, however this newborn was referred after birth to the Neonatal Care Unit located in a non-endemic area, so we consider it was indeed a case of CM.

Positive umbilical cords and neonatal peripheral blood samples from units of analysis were included for calculating the prevalence, and all complete or partial units formed the denominator. It became evident in this study that obtaining samples from these two sites was essential for diagnosing CM and determining the magnitude of this disease. If the units of analysis were divided according to sample site, a prevalence of 2.5% (2/80) and of 2.6% (3/116) would have been obtained for malaria positive samples from the umbilical cord and neonatal peripheral blood, respectively.

The prevalence of CM reported here was obtained using thick smear as the diagnostic test; however, a greater prevalence might have been found if more sensitive techniques, such as PCR, had been used. Kamwendo *et al *and, recently, Mwangoka *et al *have found in *P. falciparum *endemic regions that when using thick smears to diagnose malaria from umbilical cord samples, the frequencies range from 0.4% to 6.0%, and when PCR is used the frequencies increase to 20% to 61% for samples taken from the same site [33,34].

Low parity, fever during the last trimester of gestation, maternal malaria at birth, placental infection by *Plasmodium spp*. and low socioeconomic level have all been associated with greater CM frequency [11,35,36]. Age of pregnant mother and maternal infection by HIV have been associated with greater *Plasmodium *trans-placental transmission risk, with a higher infection frequency and placental damage [37-40]. Falade *et al *in 2002, highlighted the strong relationship between maternal parasitaemia at birth and positive placental samples with congenital infection [11].

The study identified four cases of CM, three by *P. vivax *and one by *P. falciparum*, where the mothers had malaria during delivery; placental infection was also found in the three CM cases by *P. vivax*. The other mother had *P. vivax *malaria 10 weeks before giving birth. A previous report by our group on neonatal malaria (where vertical *P. vivax *transmission was suspected), showed that two of the four positive mothers were in their first or second gestation [18]; the present study found that three (60%) of the five mothers of the CM cases were secundiparous. In contrast to other reports [18,41], the mothers of the CM cases in the present study were older than 20 years of age.

LBW has been associated with infection by *P. falciparum *during pregnancy and at birth, which is explained by a combination of intrauterine growth restriction and PB [42] in both high and low malaria transmission regions, accompanied by almost a four-fold increased risk when there has been placental infection [41]. In this study, only the newborn infected by *P. falciparum *weighed less than 2,500 grams and was born at week 30 of gestation. Such PB could have been due to the mother presenting *P. falciparum *infection at birth. Infection by this species has been associated with a greater risk of PB due to the magnitude of the induced inflammatory response. Many reports have associated LBW with MIP caused by *P. falciparum *[41], but only a few have shown the effect of the infection by *P. vivax *on birth weight. Nosten *et al *reported an increased risk of LBW associated with *P. vivax *infection [43]. The absence of LBW in CM cases of mothers with MIP by this specie was notable in this report; this could be explained because the infection occurred at the end of the gestation and the gestations went to full term. Anthropometric measurements in all newborns, including the premature case, were appropriate for the gestational age.

In low endemic and unstable transmission areas the acquisition of immunity against *Plasmodium spp*. is difficult and incomplete, so there is little passage of antibodies through the placenta [41]. In this scenario, the clinical manifestations of a CM case are usually fever accompanied by irritability, anorexia, hepatosplenomegaly, haemolytic anaemia and ichtericia [14,18,44]. In contrast, in high endemic and stable transmission regions where it is considered that the opposite is true, signs and symptoms of active infection are infrequent [45,46].

This study location had unstable transmission of malaria, which has thus hampered the induction of effective immunity against the parasite. In contrast to a previous report on cases of neonatal malaria by *P. vivax *in the same region, in which the newborns presented severe disease [18], in the present study the CM cases by this specie were asymptomatic. This difference could have been due to the infections being identified during the pre-clinical phase as a consequence of active search, and to a timely treatment.

The newborn with CM by *P. falciparum *presented a very serious disease requiring treatment in a neonatal intensive care unit outside the endemic zone. Other conditions including sepsis by *Escherichia coli*, hyaline membrane disease and PB converged in this patient and can explain the extreme severity of the clinical picture. Research in severe malaria has shown that some cases of extreme clinical severity in both children and adults can be explained by co-infection with bacterial agents [47] which, even in the case of Gram negative bacteraemia, have been reported as increasing the risk of death from malaria [48,49].

## Conclusions

1) CM is a frequent event in newborns of mothers who have had malaria during pregnancy in a coastal region of Colombia;

2) The main maternal characteristics in these cases of CM were having malaria during the last trimester or during delivery, and placental infection.

## Competing interests

The authors declare that they have no competing interests.

## Authors' contributions

JGPJ: conceiving and designing the research proposal, analysing the data and writing the manuscript; GA: analysing the data and writing the manuscript; AT: designing the investigation, coordinating the field research and critically reviewing the manuscript; MA: designing the research, acquiring the data and critically reading the manuscript; SC: acquiring the data and critically reading the manuscript; SB: conceiving and designing the research proposal, interpreting the data and writing the manuscript.

All authors read and approved the final manuscript.
